# The NYU Children’s Health and Environment Study

**DOI:** 10.1007/s10654-020-00623-6

**Published:** 2020-03-25

**Authors:** Leonardo Trasande, Akhgar Ghassabian, Linda G. Kahn, Melanie H. Jacobson, Yelena Afanasyeva, Mengling Liu, Yu Chen, Mrudula Naidu, Garry Alcedo, Joseph Gilbert, Tony T. Koshy, the NYU Children’s Health and Environment Study Team, Abigail Gaylord, Adeyinka Ajayi, Adriana Garcia, Aisha Dar, Alexis Mandon, Alice Trye, Alyssa Polak, Amit Saxena, Ana Pacheco Ochoa, Ana Ramirez, Anandini Suri, Andrea Larkin Papa, Andrea Nardello, Andrew Ailoje, Anna Kheyfets, Anne Lorraine Dumadag, Ashley Jones, Benjamin Barnett, Bettina Babu, Blanca Vargas, Bo Gu, Camille McNally, Carly Batt, Carolena Rojas Marcos, Chiemika Nwosu, Christiana Harry, Christina Awada, Christopher Haggar, Daniel Wohl, Danisha Dennie, David Fenyo, Denise Mallick, Doris Descorbeth, Douglas Donnelly, Eldad Cano, Elle Wszeborowski, Emely Tejeda, Emma Cowles, Eric Mallow, Evelyn Hernandez, Felix Le Coadic, Freddy Loffredo, Giovanna Lopez, George Nikoloudakis, Ha Young Kyung, Hai Man He, Hannah Bava, Heather Jao, Ho Fei Sit, Isabella Possagnoli, James Deats, Jamie Lee Tavarez, Jasmine Raymer, Jassly Naval, Jennifer B. Lee, Jennifer Carpio, Jessica Ospina, Jill Buyon, Jocelyn Majano, Jose Lugo Eslava, Joseline M Cruz Vasquez, Juan Pablo Robayo, Julia Malits, Kasandra Cisneros, Kayla Rae Farrell, Kirtan Kaur, Kristine Karibandi, Kevin Mendoz, Lauren Burdine, Liliany Nigam, Lillian Walton Masters, Lisa Nathan, Lonny Dym, Makhethe Mpoti, Manuel Hernandez, Maria del Mar Colon, Maria Sanchez Garcia, Marina Rosado, Mary Jo Messito, Matthew Shong, Meera T, Melissa Robbins, Michael Ferro, Michelle Gorchynski, Miriam Peters, Miriam Woodward, Nader Daoud, Nathalia Schettino, Onassis Castillo Ceballo, Paridhi Bhargava, Peggy Hsieh, Pema Sherpa, Peter Izmirly, Rachel Marconi, Rakan Alqaqaa, Ranga Bharadwaj, Rifat Iqbal, Robert Clancy, Roberta Scheinmann, Rummanu Yeasin, Sara Brubaker, Sara Long, Sarah Lazaros, Sarah Watson, Yuyan Wang, Sarvani Ramcharran, Shikha Chandarana, Shilpi Mehta-Lee, Shivani Karthikeyan, Simran Sahansra, Stefani Yanez, Stephanie Vazquez, T Meera, Tatiana Wilson, Teresa Attina, Terri-Anne Bennet, Yutian Mu

**Affiliations:** 1 Department of Pediatrics, New York University School of Medicine, New York, NY USA; 2 Department of Environmental Medicine, New York University School of Medicine, New York, NY USA; 3 Department of Population Health, New York University School of Medicine, New York, NY USA; 4 NYU Wagner School of Public Service, New York, NY USA; 5 NYU College of Global Public Health, New York, NY USA

**Keywords:** Endocrine disrupting chemicals, Obesity, Fetal growth, Epigenetics, Metabolomics

## Abstract

The aims of the NYU Children’s Health and Environment Study (CHES) are to evaluate influences of prenatal non-persistent chemical exposures on fetal and postnatal growth and pool our data with the US National Institutes of Health Environmental influences on Child Health Outcomes (ECHO) Program to answer collaborative research questions on the impact of the preconceptual, prenatal, and postnatal environment on childhood obesity, neurodevelopment, pre/peri/postnatal outcomes, upper and lower airway outcomes, and positive health. Eligible women were ≥ 18 years old, < 18 weeks pregnant, had a pregnancy that is not medically threatened, and planned to deliver at NYU Langone Hospital—Manhattan, Bellevue Hospital, or NYU Langone Hospital—Brooklyn. Between March 22, 2016 and April 15, 2019, we recruited 2469 pregnant women, from whom 2193 completed an initial questionnaire and continued into NYU CHES. Of the 2193, 88 miscarried, 28 terminated, and 20 experienced stillbirth, while 57 were lost to follow up. We report here demographic and other characteristics of the 2000 live deliveries (2037 children), from whom 1624 (80%) consented to postnatal follow-up. Data collection in pregnancy was nested in clinical care, with questionnaire and specimen collection conducted during routine prenatal visits at < 18, 18–25, and > 25 weeks gestation. These have been followed by questionnaire and specimen collection at birth and regular postpartum intervals.

## Introduction

The unique vulnerability of children to environmental hazards has been documented in many scientific studies and government reports, including a landmark 1993 US National Academy of Sciences report on pesticide exposures [1]. Yet, studies of children’s health have failed to account fully for the range of environmental influences in pregnancy, and the postnatal period that can substantially influence health from childhood through adult life. The Developmental Origins of Health and Disease hypothesis was first formulated by Barker and colleagues in the context of nutritional influences [2–5]. Nonetheless, it is widely accepted that biological, psychosocial, chemical, and physical exposures are equally influential [6].

Until now, progress toward elucidating the role of the environment in childhood obesity and other chronic conditions has been slow and incremental. Most studies have examined relatively small populations of children [6]; have considered only one chemical exposure at a time; have had little statistical power to examine interactions among chemical, social, and behavioral factors; and have had limited ability to examine gene–environment interactions [7]. Little is known about possible interactions and synergies among chemicals or between chemicals and other environmental hazards, even though the environment of a child includes mixtures of chemical and biological toxicants. Gene-environment interactions and epigenomic effects of exposures are just beginning to be explored.

For example, the recent explosive increase in the prevalence of obesity reflects a complex interplay among (1) changes in individual behaviors; (2) changes in community structure, lifestyle, and the “built environment”; and (3) exposures to certain synthetic chemicals (e.g., endocrine disruptors) that might disrupt energy balance [6]. Control of the obesity epidemic will require understanding each of these factors and the interplay among them. While previous cohort studies have contributed greatly to identifying many individual-level factors that contribute to the development of obesity in children and its persistence into adulthood in the US and other countries [8–21], these studies have several limitations [6]:

- Previous studies have not fully capitalized on the life-course approach to chronic disease epidemiology [22].
- Although some studies have collected genetic data on participants and been able to identify polymorphisms that increase the risk of obesity, they have not simultaneously collected the data on environmental exposures needed to carefully examine the interactions of genetic and environmental factors with diet, physical activity, or epigenetic changes—all of which might predict risk of obesity.
- Many prior cohorts have been limited in their capacities to identify risk factors for obesity that may be unique among Hispanics, a population for which obesity prevalence is increasing especially rapidly [23, 24].
- Past studies have not assessed features of the built environment that encourage healthy diet and physical activity among children living in urban areas [25].

In the context of the broad array of environmental stressors, increasing human and laboratory evidence suggests that exogenous chemicals influence developmental metabolic programming and provoke oxidative stress, a major pathophysiologic mechanism that underlies cardiometabolic risks [26]. These include (1) phthalates (used to soften plastics and as scents), which increase expression of peroxisome proliferator-activated receptors [27] that play key roles in lipid and carbohydrate metabolism [28]; (2) bisphenols (found in aluminum can linings and thermal paper receipts), which are mildly estrogenic, increase fat in adipocytes [29], and disrupt pancreatic β-cell function; (3) polycyclic aromatic hydrocarbons (PAHs, present in air pollution), which promote inflammation and increase visceral fat in animal models [30, 31]; and (4) organophosphate pesticides (OPs), which are thyroid hormone antagonists that contribute to pre-diabetes, lipid metabolism abnormalities, and obesity in animals [32].

Longitudinal studies of prenatal exposure in humans, especially for phthalates and bisphenols, have not yielded expected findings [33–37]. This might be attributed to (1) limited exposure assessment via collection of a limited number of spot samples during later pregnancy, which restricts insight into how the effects of exposure depend on the earliest stages of fetal development; (2) lack of fetal growth data to evaluate intrauterine effects; (3) use of body mass index (BMI) rather than specific measures of fat mass; (4) failure to measure replacements of bisphenol A and di-2-ethylhexylphthalate, or DEHP (particularly bisphenol S, or BPS, diisononylphthalate, or DINP, and diisodecylphthalate, or DIDP) which have begun to be used over the past decade; and (5) lack of mechanistic insight.

The purpose of this manuscript is to describe the NYU Children’s Health and Environment Study (CHES), which was designed to overcome these limitations and identify environmental and genetic causes of normal and abnormal growth, development, and health from fetal life onward.

## Scope of research

The general aims of NYU CHES are to:

- Evaluate influences of prenatal non-persistent chemical exposures on fetal and postnatal growth.
- Evaluate prenatal non-persistent chemical exposures in relation to epigenetic marks and gene expression.
- Identify metabotypes related both to exposures and cardiometabolic outcomes, and assess exposures and metabotypes in relation to oxidative stress and perturbations of adiponectin, leptin, and sex hormones.
- Pool our data with that of other cohorts in the US National Institutes of Health (NIH) Environmental influences on Child Health Outcomes (ECHO) program and answer collaborative research questions on the impact of the preconceptual, prenatal, and postnatal environment on childhood obesity, neurodevelopment, pre/peri/postnatal outcomes, upper and lower airway outcomes, and positive health.

## Study population and design

### Overview

NYU CHES is a clinically enrolled, prospective cohort study from fetal life onward. Since March 2016, NYU CHES staff enrolled pregnant women into a biobank study from three NYU Grossman School of Medicine affiliates: NYU Langone Hospital—Manhattan, Bellevue Hospital, and NYU Langone Hospital—Brooklyn, diverse hospitals serving a wide array of populations. Formerly known as Tisch Hospital, NYU Langone Hospital—Manhattan is a major acute care center for the New York City metropolitan area. Bellevue is the flagship hospital of the largest municipal hospital system in North America (the New York City Health and Hospitals Corporation). NYU Langone Hospital—Brooklyn’s Family Health Center is the second largest federally qualified health center in the nation.

### Eligibility and enrollment

Eligible women were ≥ 18 years old, < 18 weeks pregnant, had a pregnancy that was not medically threatened, and planned to deliver at one of the study hospitals. Study staff were bilingual and study materials were available in English, Spanish, and Chinese. To proceed from the biobank study into NYU CHES, participants must have completed an initial questionnaire collecting sociodemographic data and medical history, as well as behaviors, exposures, and experiences in the first trimester.

## Study cohort

### Pregnant women and their children

**Fig. 1:**
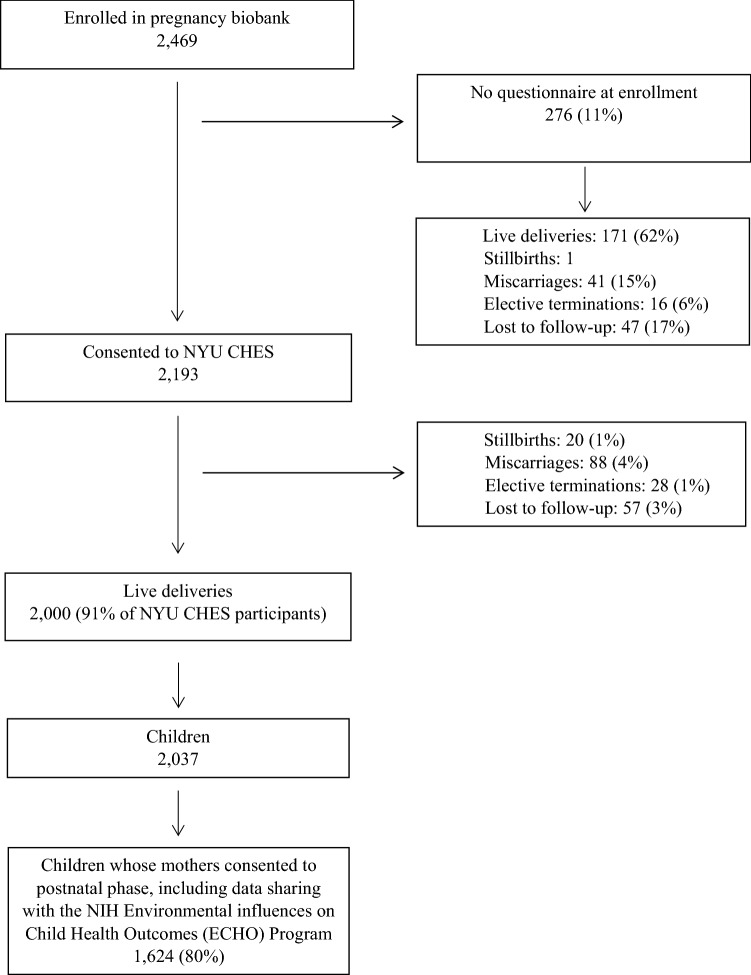
NYU Children’s Health and Environment Study (NYU CHES): first 2000 births

Between March 22, 2016 and April 15, 2019, we enrolled 2469 pregnant women into a pregnancy biobank, of whom 2193 women completed a questionnaire and continued into NYU CHES (Fig. 1). From 276 pregnant participating in the biobank, who were not enrolled in NYU CHES because of no questionnaire at enrollment (despite all our efforts), we are aware of 171 live births, while 16 electively terminated and 1 had a stillbirth. Early miscarriage may have contributed to the lack of a questionnaire in 41 of the 276. These represent an important population to study together with miscarriages in women who did complete a questionnaire (n = 88, see below), as environmental exposures in early pregnancy may have differential effects on earlier as opposed to later miscarriage among those who completed questionnaires. Of 2193 pregnant women who continued into NYU CHES, 88 miscarried (mentioned above), 28 terminated, and 20 experienced stillbirth, while 57 were lost to follow up. The 2000 live deliveries resulted in 2037 children; the mothers of 1624 (80%) of these children consented to join the postnatal phase of the study and gave permission to share their children’s identifiable data with the ECHO program.

The cohort is multiracial, multiethnic, and has substantial socioeconomic diversity (Table 1). The NYU Langone—Manhattan mothers are older, less likely to be Hispanic, more likely to have private health insurance, more likely to be employed, and more likely to be married or partnered; have higher income and education; and have lower BMI than the participants from the other two study sites. Across all recruitment locations, the vast majority are married or partnered and never smoked. While Manhattan mothers were more likely to have used alcohol prior to pregnancy (20%), 10% of participants from two other recruitment locations continued to use alcohol during pregnancy.

**Table 1:** Descriptive characteristics of NYU CHES study participants by recruitment location

Characteristic^a^	NYU Langone Hospital-Manhattan	NYU Langone Hospital-Brooklyn	Bellevue Hospital	Total NYU CHES
	n = 1076	n = 673	n = 444	n = 2193
Age at enrollment, mean (SD), years	33.9 (4.5)	29.3 (5.8)	30.2 (5.9)	31.8 (5.6)
Race/ethnicity, n (%)				
Hispanic	169 (16%)	563 (84%)	339 (77%)	1071 (49%)
White, not of Hispanic origin	652 (61%)	42 (6%)	33 (8%)	727 (33%)
Black, not of Hispanic origin	62 (6%)	33 (5%)	35 (8%)	130 (6%)
Asian	143 (13%)	27 (4%)	23 (5%)	193 (9%)
Other	12 (1%)	4 (1%)	4 (1%)	20 (1%)
Multiple	36 (3%)	2 (< 1%)	6 (1%)	44 (2%)
Marital status, n (%)				
Married/living with a partner	1024 (95%)	543 (81%)	344 (78%)	1911 (88%)
Divorced/separated	10 (1%)	23 (3%)	15 (3%)	48 (2%)
Single/widowed	38 (4%)	104 (16%)	84 (19%)	226 (10%)
Education, n (%)				
High school or less	33 (3%)	434 (66%)	237 (54%)	704 (32%)
Some college but no degree	64 (6%)	110 (17%)	82 (19%)	256 (12%)
Associate degree	31 (3%)	43 (6%)	32 (7%)	106 (5%)
Bachelor’s degree	380 (36%)	55 (8%)	63 (15%)	498 (23%)
Post graduate degree	559 (52%)	20 (3%)	23 (5%)	602 (28%)
Household income, n (%)				
Less than $30,000	24 (2%)	196 (30%)	142 (34%)	362 (17%)
$30,000–$49,999	36 (4%)	78 (12%)	64 (15%)	178 (8%)
$50,000–$74,999	90 (8%)	31 (5%)	20 (5%)	141 (7%)
$75,000–$99,999	93 (9%)	6 (1%)	7 (2%)	106 (5%)
$100,000 or more	778 (74%)	18 (3%)	9 (2%)	805 (38%)
Don’t know	34 (3%)	323 (49%)	172 (42%)	529 (25%)
Employed, n (%)	916 (86%)	271 (41%)	225 (51%)	1412 (65%)
Insurance, n (%)				
Public	139 (13%)	587 (88%)	413 (96%)	1139 (52%)
Private	934 (87%)	83 (12%)	15 (4%)	1032 (48%)
Use of cigarettes and other related products^b^ during pregnancy, n (%)				
Never	991 (92%)	607 (90%)	385 (87%)	1983 (91%)
Used, stopped in pregnancy	72 (7%)	57 (9%)	51 (11%)	180 (8%)
Used, continued in pregnancy	10 (1%)	9 (1%)	8 (2%)	27 (1%)
Alcohol use during pregnancy, n (%)				
Never	155 (15%)	368 (55%)	211 (48%)	734 (33%)
Used, stopped in pregnancy	700 (65%)	234 (35%)	191 (43%)	1125 (52%)
Used, continued in pregnancy	215 (20%)	66 (10%)	39 (9%)	320 (15%)
Pre-pregnancy body mass index, mean (SD), kg/m^2^	24.7 (5.0)	27.5 (5.5)	28.6 (6.5)	26.3 (5.7)
Nulliparous, n (%)	687 (64%)	227 (34%)	203 (46%)	1117 (51%)

^a^Missing data: race/ethnicity: 8 (< 1%); marital status: 8 (< 1%); education: 27 (1%); household income: 72 (3%); insurance: 22 (1%); cigarettes and other related prodcuts use: 3 (< 1%); alcohol use: 14 (1%); pre-pregnancy body mass index: 27 (1%); employment: 14 (1%); parity: 6 (< 1%)

^b^Includes cigars, cigarillos, hookah, patches, gums, and other nicotine products

To evaluate whether study participants are representative of their respective hospital populations, we compared their aggregate demographic characteristics to those of all patients seen during the same time period at NYU Langone—Manhattan and Brooklyn, the two recruitment locations for which we had hospital-wide information (Table 2). Data for all patients who had their first prenatal visit at these two locations between March 22, 2016 and April 15, 2019 and met the study eligibility criteria were aggregated. Because we did not approach every patient who received prenatal care at these sites, the percentage of study participants should not be misinterpreted as a participation rate. Descriptive data suggest that maternal age, race and ethnicity, insurance status, and parity were nearly identical among participants compared to the overall clinic populations. Participants appeared to be more likely to be married, however, compared to the overall population of pregnant women who visited these clinical sites.

**Table 2:** Representativeness of NYU CHES participants from NYU Langone—Manhattan and Brooklyn

Characteristic	NYU Langone Obstetrics & Gynecology Associates (New York, NY)		Sunset Park Family Health Center at NYU Langone, Women’s Health (Brooklyn, NY)	
	All patients seen in the clinic	NYU CHES participants	All patients seen in the clinic	NYU CHES participants
	n = 8176	n = 1076	n = 2723	n = 673
Age at first prenatal visit, mean (SD), years	34.1 (5.1)	33.9 (4.5)	29.9 (6.1)	29.3 (5.8)
Race/ethnicity, n (%)				
Hispanic	766 (10%)	169 (16%)	1988 (74%)	563 (84%)
White, not of Hispanic origin	5106 (65%)	652 (61%)	223 (8%)	42 (6%)
Black, not of Hispanic origin	538 (7%)	62 (6%)	147 (5%)	33 (5%)
Asian	972 (13%)	143 (13%)	111 (4%)	27 (4%)
Other/multiple	425 (5%)	48 (4%)	237 (9%)	6 (1%)
Missing	369	2	17	2
Marital status, n (%)				
Married/living with a partner	5279 (77%)	1024 (95%)	1131 (46%)	543 (81%)
Divorced/separated	60 (1%)	10 (1%)	30 (1%)	23 (3%)
Single	1451 (21%)	38 (4%)	1283 (52%)	104 (16%)
Other	41 (1%)	0	9 (< 1%)	0
Missing	1345	4	270	3
Nulliparous, n (%)	4939 (62%)	687 (64%)	1096 (40%)	227 (34%)
Missing	167	0	13	2
Insurance, n (%)				
Public	1112 (14%)	139 (13%)	2366 (87%)	587 (88%)
Private	6960 (86%)	934 (87%)	341 (13%)	83 (12%)
Missing	104	3	16	3
Pre-pregnancy BMI, mean (SD), kg/m^2^	24.8 (5.2)	24.7 (5.0)	28.1 (6.1)	27.5 (5.5)
Missing	3509	3	1739	15
Gestational age at delivery, mean (SD), weeks^a^	38.8 (2.6)	39.0 (1.7)	38.9 (2.0)	39.0 (1.8)
Missing	1930	23	816	12
Child sex, male, n (%)^b^	3256 (51%)	534 (53%)	980 (51%)	311 (51%)
Missing	40	27	11	11
Birth weight, mean (SD), g^b^	3215 (607)	3226 (553)	3052 (811)	3279 (536)
Missing	1859	52	2534	30

^a^Among live deliveries

^b^Among live children

To compare NYU CHES births with those in all of New York City, we obtained aggregate-level data on women who gave birth in New York City in 2016 and who sought prenatal care in the first trimester using publicly available data from the Bureau of Vital Statistics at the New York City Department of Health and Mental Hygiene [38]. Comparison of NYU CHES live births to the population of births in New York City reveals substantial similarity, supporting the broader generalizability of findings from NYU CHES (Table 3). Although NYU CHES participants with live births are substantially more likely to be Hispanic, less likely to be Asian or Non-Hispanic Black, and more likely to be married than those who gave birth across New York City, distributions of maternal age, education, parity, and pre-pregnancy BMI category are similar, as are their children’s delivery method, gestational age at birth, sex, and birth weight.

**Table 3:** Comparison of NYU CHES participants with women who delivered live births in New York City, 2016

Characteristic	2016 New York City birth data	NYU CHES participants with live deliveries
	n = 88,924	n = 2000
Mother’s age, years		
< 20	1754 (2%)	45 (2%)
20–24	11,977 (14%)	217 (11%)
25–29	22,442 (25%)	425 (21%)
30–34	28,737 (32%)	732 (37%)
35–39	18,850 (21%)	454 (23%)
40+	5164 (6%)	127 (6%)
Race/ethnicity, n (%)		
Hispanic	23,614 (27%)	965 (48%)
Asian/Pacific Islander	16,586 (18%)	180 (9%)
Non-Hispanic White	33,737 (38%)	677 (34%)
Non-Hispanic Black	13,843 (16%)	114 (6%)
Other	1096 (1%)	59 (3%)
Missing	48	5
Marital status, n (%)		
Married	59,466 (67%)	1757 (88%)
Not married	29,458 (33%)	242 (12%)
Missing	0	1
Education, n (%)		
High school or less	31,321 (35%)	635 (32%)
Some college, associate degree	18,914 (21%)	327 (16%)
Bachelor’s degree	20,864 (24%)	457 (23%)
Post graduate degree	17,646 (20%)	564 (29%)
Missing	179	17
Nulliparous, n (%)	38,772 (44%)	1002 (50%)
Missing	1	3
Pre-pregnancy BMI category, n (%)		
Underweight	4920 (5%)	41 (2%)
Normal	48,515 (55%)	947 (48%)
Overweight	21,091 (24%)	571 (29%)
Obese	14,191 (16%)	422 (21%)
Missing	207	19
Multiple births, n (%)	3250 (4%)	37 (2%)
Delivery method, n (%)		
Vaginal	59,366 (67%)	1264 (67%)
Cesarean section	29,558 (33%)	615 (33%)
Missing	0	121
Gestational age category, n (%)		
17–31 weeks	1222 (1%)	14 (1%)
32–33 weeks	912 (1%)	18 (1%)
34–36 weeks	5686 (7%)	124 (6%)
37–47 weeks	81,104 (91%)	1797 (92%)
Missing	0	47
Preterm births (< 37 weeks), n (%)	7820 (9%)	156 (8%)
Missing	0	47
Child sex, male, n (%)^a^	45,423 (51%)	1031 (52%)
Missing	0	56
Birth weight category, n (%)^a^		
< 1000	521 (1%)	6 (< 1%)
1000–1499	649 (1%)	9 (< 1%)
1500–1999	1328 (1%)	34 (2%)
2000–2499	4613 (5%)	101 (5%)
2500–2999	17,852 (20%)	389 (20%)
3000–3499	35,977 (40%)	777 (40%)
3500–3999	22,295 (25%)	490 (26%)
4000–4499	5027 (6%)	105 (5%)
4500–4999	605 (1%)	17 (1%)
5000+	57 (< 1%)	1 (< 1%)
Missing	0	108
Low birth weight (< 2500 g), n (%)^a^	7111 (8%)	150 (8%)
Missing	0	108

^a^NYU CHES: among live children, n = 2037

From 2193 pregnant participating in NYU CHES, 20 women had stillbirths, 28 had elective terminations, and 88 experienced miscarriages. The cohort includes 37 multiple births (Table 4). Cesarean section rates were comparable at the two NYU Langone hospitals, and higher than at Bellevue (32–37% vs. 22%). Preterm birth was higher at Bellevue versus the other sites (10% vs. 7–8% for preterm birth), in keeping with known associations of sociodemographic factors with preterm birth [39, 40]. Nonetheless, low birth weight was the lowest in NYU Brooklyn (6%) compared to NYU Manhattan and Bellevue (9 and 8%, respectively).

**Table 4:** Birth outcomes in the initial phase of NYU CHES

Characteristic^a^	NYU Langone Hospital—Manhattan	NYU Langone Hospital—Brooklyn	Bellevue Hospital	Total
	n = 1076	n = 673	n = 444	n = 2193
Pregnancy outcome, n (%)				
Live delivery	1006 (95%)	611 (93%)	383 (90%)	2000 (94%)
Stillbirth	9 (1%)	7 (1%)	4 (1%)	20 (1%)
Miscarriage	28 (3%)	33 (5%)	27 (6%)	88 (4%)
Elective termination	11 (1%)	5 (1%)	12 (3%)	28 (1%)
Multiple births (all twins), n (%)^b^	24 (2%)	9 (1%)	4 (1%)	37 (2%)
Gestational age at delivery, mean (SD), weeks^b^	39.0 (1.7)	39.0 (1.8)	39.0 (2.0)	39.0 (1.8)
Preterm births (< 37 weeks), n (%)^b^	79 (8%)	40 (7%)	37 (10%)	156 (8%)
Delivery method, n (%)^b^				
Vaginal	591 (63%)	394 (68%)	279 (78%)	1264 (67%)
Cesarean section	352 (37%)	186 (32%)	77 (22%)	615 (33%)
Child sex, n (%), male^c^	534 (53%)	311 (51%)	186 (50%)	1031 (52%)
Birth weight, mean (SD), g^c^	3226 (553)	3279 (536)	3284 (571)	3284 (568)
Low birth weight (< 2500 g), n (%)^c^	86 (9%)	36 (6%)	28 (8%)	150 (8%)

^a^Missing data: pregnancy outcome: 57 (3%); gestational age at delivery and preterm births: 47 (2%), delivery method: 121 (6%); child sex: 56 (3%); birth weight and low birth weight: 108 (5%)

^b^Among live deliveries

^c^Among live children

## Data collection in the prenatal phase

Visits in pregnancy were nested in clinical care, with questionnaire and specimen collection conducted during three routine prenatal visits in the following intervals: < 18 weeks; 18–25 weeks; and > 25 weeks gestation. These were followed by visits at birth and at regular postpartum intervals (which occur separately from clinical care). Maternal blood, urine, and saliva samples were collected at each prenatal visit, vaginal samples were collected at least once in pregnancy, and fecal samples were collected in a subsample postnatally. At birth, cord blood samples included whole blood, serum, plasma, and PAXgene tubes for RNA analysis. Placental cores (2 × 2 cm) and segments of the umbilical cord were also collected. Maternal urine samples were available for all three time points during pregnancy in a majority of participants, and nearly four-fifths of participants had placental and/or cord blood samples available (Table 5).

**Table 5:** Questionnaire and prenatal/neonatal specimen availability by time point, live deliveries (n = 2000)

	During pregnancy gestational age, weeks			Any time during pregnancy	At birth
	< 18	18–25	> 25		
Questionnaires, n (%)	2000 (100%)	1726 (86%)		2000 (100%)	1080 (54%)
Diet History Questionnaire II, n (%)		1406 (70%)		1406 (70%)	
Maternal urine, n (%)	1777 (89%)	1318 (66%)	1627 (81%)	1938 (97%)	
Maternal saliva, n (%)	1603 (80%)	1074 (54%)	1458 (73%)	1868 (93%)	
Maternal whole blood, n (%)	1146 (57%)	479 (24%)	850 (43%)	1514 (76%)	
Maternal PAXgene RNA tube, n (%)	1174 (59%)		850 (43%)	1441 (72%)	
Maternal plasma, n (%)		477 (24%)		477 (24%)	
Maternal serum, n (%)	1155 (58%)	492 (25%)	858 (43%)	1521 (76%)	
Maternal vaginal swab, n (%)	1088 (54%)	860 (43%)	1173 (59%)	1491 (75%)	
Placental core, n (%)					1509 (75%)
Umbilical cord, n (%)					1491 (75%)
Cord blood (PAXgene RNA tube), n (%)					1392 (70%)
Cord blood (Whole), n (%)					1059 (53%)
Cord serum, n (%)					886 (44%)
Cold plasma, n (%)					417 (21%)
Neonatal meconium, n (%)					396 (20%)

### Questionnaires and medical chart abstraction

Questionnaires administered in the first two pregnancy intervals and at birth collected information on participant and partner demographics as well as participants’ reproductive health and history, medication and substance use, employment, address, and home life (Table 6). The questionnaires included a variety of validated psychosocial scales (the Pregnancy-Related Anxiety Scale [41] and the Patient Health Questionnaire-9 (PHQ-9) [42] for measurement of depression). Women were also asked about their sleep during pregnancy using the Pittsburgh Sleep Quality Index (PSQI) [43]. Once during pregnancy, participants completed the Diet History Questionnaire II (DHQ II), a publicly available food frequency questionnaire developed by the US National Cancer Institute [44]. Of the four versions of the DHQ, we used the version that asks about diet in the past year, including portion size (available and validated in English and translated to Spanish). Maternal physical activity was assessed in all three intervals during pregnancy using the International Physical Activity Questionnaire-Short Form (IPAQ) [45].

**Table 6:** Variables measured in NYU CHES through child age 2 years

	< 18 weeks	18–25 weeks	> 25 weeks	Birth	4–7 months	8–11 months	12–17 months	18–23 months	24–35 months
Maternal/family characteristics									
Family sociodemographic factors	Q, EHR	EHR			Q	Q	Q	Q	Q
Gestational hypertension, preeclampsia and gestational diabetes	Q, EHR	Q, EHR	EHR	Q	Q				
Current pregnancy information, including morning sickness and vitamin/medication use	Q	Q		Q					
Maternal stress, depression, or anxiety	Q	Q	Q		Q			Q	Q
Adverse events during pregnancy	EHR	EHR	EHR	Q					
Reproductive history	Q, EHR	EHR	EHR						
General health and health history	Q, EHR	Q, EHR		Q, EHR				Q	Q
Sleep	Q	Q		Q					
Physical activity	Q	Q	Q	Q					
Maternal substance use	Q	Q		Q	Q	Q	Q	Q	Q
Housing characteristics	Q	Q		Q				Q	
Home environment								Q	
Pets and pests	Q	Q		Q	Q	Q	Q	Q	
Maternal social support, exposure to domestic violence		Q						Q	Q
Diet		Q							
Labor and delivery of previous birth, and previous pregnancies	Q, EHR				Q, EHR				
Maternity leave/employment	Q	Q		Q	Q				
Childcare					Q	Q	Q	Q	
Food insecurity	Q				Q				
Parenting and family cohesion									Q
Child characteristics									
Diet and feeding					Q	Q	Q	Q	
General health, medications, and hospitalizations					Q, EHR	Q, EHR	Q, EHR	Q, EHR	Q, EHR
Early development					Q	Q	Q	Q	Q
Language development									Q
Emotional and behavioral development							Q		Q
Floor time and devices that hold the baby					Q	Q	Q		
Media exposure					Q	Q	Q	Q	Q
Sleep					Q	Q	Q	Q	
Clinical measures									
Fetal sonographic measurements	Measured	Measured	Measured						
Brachial artery distensibility, blood pressure								M:Measured	M:Measured C:Measured
Weight and height	M:Q, EHR P:Q	M:EHR	M:EHR	M:Q, EHR C:Q, EHR	C:Q	C:Q	C:Q	M:Measured C:Measured	M:Measured C:Measured
Anogenital distance				Measured				Measured	Measured
Fat, lean mass (DXA scan)								C:Measured	C:Measured
Biological measures									
Bisphenols, phthalate metabolites, dialkylphosphate metabolites, polycyclic aromatic hydrocarbon metabolites	M:Urine	M:Urine	M:Urine					C:Urine	C:Urine
Biomarkers of oxidant stress	M:Urine	M:Urine	M:Urine					C:Urine	C:Urine
Metabolomics	M:Urine	M:Urine	M:Urine					C:Urine	C:Urine
Adiponectin and leptin				Cord serum					
Thyroid function and Thyroid peroxidase antibody	M:Serum								
Biomarkers of inflammation and endothelial dysfunction	M:Serum	M:Serum	M:Serum	Cord serum					
Sex steroids			M:Serum						
Methylomics (genome-wide)				Cord PAXgene					
Transcriptomics (genome-wide)				Cord PAXgene					

*Q* questionnaire as completed by mothers, *EHR* electronic health record, *C* child, *M* maternal, *P* partner

Substantial data are obtained directly from electronic health records, including maternal age at enrollment, parity/gravity, gestational hypertension, preeclampsia, weight and blood pressure at each prenatal visit, and mode of delivery. Gestational diabetes will be evaluated using the American College of Obstetrics and Gynecology guidelines for glucose tolerance testing. Fetal growth data and estimated gestational dating collected during regular ultrasounds are also extracted from electronic health records, including crown-rump length, biparietal diameter, head circumference, transverse cerebellar diameter, femur length, and abdominal circumference, as appropriate (Table 6).

### Specimen analyses

With ECHO support, we are analyzing urine at each time point in pregnancy for OP metabolites, phthalate metabolites, bisphenols, PAHs, biomarkers of oxidative stress (F2-isoprostane and 8-hydroxydeoxyguanosine), cotinine, creatinine, and metabolomic indicators (Table 6). We are analyzing adiponectin and leptin in cord blood, as well as cytokines in serum at each pregnancy time point and in cord blood. Thyroid function (thyroid stimulating hormone, free and total thyroxine, free and total triiodothyronine, and thyroid peroxidase antibody) is being measured in serum samples collected at < 18 weeks, and maternal sex hormones (fractionated estrogens, free and total testosterone, estradiol, estrone, estriol, sex hormone binding globulin, and dehydroepiandrosterone) are being measured in the third pregnancy interval. Genome-wide methylome and transcriptome analyses will be performed on cord blood samples.

## Data collection in the postnatal phase

ECHO also supports the postnatal phase of the study through age 2 years with in-person assessments that occur at ages 12–23 and 24–35 months outside of regular medical care. Each visit is coupled with a comprehensive questionnaire filled out by mothers prior to or during the visit. In addition, evaluations include questionnaires administered online or via phone at 4–7, 8–11, and 18–23 months (Table 6). Mothers are encouraged to use the information from visits with their primary care providers to report on children’s anthropometric measures in every questionnaire. They also report on history of hospitalizations and illnesses. Questionnaires evaluate breastfeeding and infant feeding history, sleep patterns, childcare, and media exposures. Child early development is assessed using the Ages and Stages Questionnaire–Third Edition (ASQ–3) [46] and the World Health Organization questionnaire on achievement of major gross motor milestones [47]. Parental rating of the child’s behavior is assessed at 12–17 months using the Brief Infant–Toddler Social and Emotional Assessment (BITSEA) [48], and at 24–35 months using the Child Behavior Checklist (CBCL) [49]. Children’s language development is assessed using the Language Development Survey (LDS) at age 24–35 months [49]. When the children are 24–35 months, we obtain information on parenting using the Parenting Scale [50] and family relationship using the Family Environment Scale (FES) [51].

In-person visits at ages 12–23 and 24–35 months take approximately 60 min and include collection of child urine samples and anthropometric measures; a dual-energy X-ray absorptiometry scan to evaluate bone, fat and muscle mass; and measurement of anogenital distance, a well-known proxy for sex steroid exposure in utero [52]. With ECHO support, 12–23 and 24–35 month urine samples will be assayed for the same analytes as the prenatal samples. During these in-person visits, we also ask mothers to complete a 24-hour recall of their child’s food intake using the Automated Self-Administered 24-hour Dietary Assessment Tool (ASA—24). Maternal depression is assessed via the Edinburgh Postnatal Depression Scale (EPDS) in the 4–7 month questionnaire and by the Brief Symptoms Inventory (BSI) in the 18–23 month questionnaire [53, 54]. At age 24–35 months, maternal stress is assessed using the Perceived Stress Scale (PSS) [55]. Food insecurity is assessed at 4–7 months using the US Department of Agriculture’s Core Food Insecurity Module.

Parents also consent to prospective, passive data collection from the electronic health record for pediatric visits. This includes anthropometric measurements; outpatient medical diagnoses; medications/immunizations; laboratory values including hemoglobin and blood lead; and inpatient and emergency room visit records, including diagnoses, procedures, and medications.

Because NYU CHES is a participating cohort in the NIH ECHO Program, our data collection is being aligned and supplemented, as necessary, to fulfill the ECHO-wide Cohort Data Collection Protocol (EWCP). Harmonized data will then be consolidated across the 70 ECHO cohorts nationally, comprising a sample of at least 50,000 children. This will enhance the statistical power and generalizability of findings related to environmental and preventable predictors of childhood disease and disability. The EWCP includes measures of neurodevelopment, asthma, obesity, pre-, peri-, and immediate postnatal outcomes, as well as positive health. To align our cohort with the EWCP and ensure its completion as children evolve through life stages beyond infancy, we have planned follow-up visits at 36–59 and 60–83 months of age.

## Data management and statistical power

### Data preparation

The questionnaire data are collected and managed using REDCap electronic data capture tools hosted at NYU Langone Health [56, 57]. An extensive set of validation rules and skip logic are implemented to ensure data quality. Data collected by measurements are recorded in REDCap or downloaded from the specific softwares to a secure location on the study server. Information from participants’ electronic health records is obtained from EPIC (all sites) and QuadraMed (Bellevue). The data from each data source are checked and cleaned. All missing data, implausible values, logical errors, and outliers are reviewed. Then, the data from all sources are harmonized and consolidated.

### Privacy protection

The study data are securely stored on the NYU Langone Health network behind a firewall. The data are accessible exclusively to the NYU CHES staff authorized by the Principal Investigator. A set of physical, technical, and administrative controls are implemented to ensure data protection. Data access rights are assigned to personnel according to their role in the study.

The links between participants’ unique identification numbers and potentially identifying and protected health information (PHI) are securely stored on the NYU Langone Health server and are accessible only to authorized study personnel. With participant permission, specimens will be banked indefinitely with identifying links. A data transfer agreement is required for release of any data to external investigators. Depending on the Institutional Review Board approval stipulations, the datasets for analyses are either de-identified or limited.

### Statistical power

Power analyses are presented in Table 7 and summarize minimally detectable difference in the unit of standard deviation (SD) for a continuous outcome between exposed and unexposed groups according to various exposure prevalences. For example, when 10% of the cohort are the exposured, the NYU CHES full cohort with the targeted 2000 live births will have 80% power to detect a difference as small as 0.209 SD between the exposure groups at a type I error level of 5% using a two-sample *t* test. Considering the exposure variable as continuous variable, the sample size of 2000 will have 80% power to detect a change of 0.063 SD in the outcome for one SD increase in the exposure variable. This effect size translates into 0.13 BMI in children, which is much smaller that the main effect previously reported for other environmental exposures such as dietary factors [58]. Note that many outcome variables will be repeatedly measured in NYU CHES, and this can lead to increased statistical power to study exposure effects. Minimally detected odds ratios for 20% exposure when the incidence in the exposed group is 5% or 10% are 1.65 and 1.96, respectively, in the CHES full cohort (n = 2000), and these minimum detectable ORs will be improved to 1.34 and 1.24 for one SD increase in continuous exposure variable. For gene-environment interaction analysis, power analyses were performed assuming an additive mode of inheritance for the gene. With a type I error level of 5% and the sample size of 2000, and the main effect of gene set at 0.20 SD increment of outcome per SD change in the exposure and the main effect of environment at 0.05 SD increment of outcome per SD change in the exposure, we have 80% power to detect minimal detectable interaction effect of 0.09, if the allele frequency is 40%. For allele frequencies equal to 30%, 20%, and 10%, the minimal detectable interaction effects will be 0.10, 0.11, and 0.15, respectively.

**Table 7:** Minimally detectable difference in standard deviation between groups according to exposure prevalence

Proportion exposed (%)	NYU CHES cohort (n = 2000)
50	0.125
20	0.157
10	0.209
5	0.287
1	0.630

The minimally detectable difference in the unit of standard deviation of outcome between exposed versus unexposed group were estimated using two-sample *t* test assuming equal variance with type I error of 5% at power of 80%

## Strengths and limitations

A leading strength of the cohort is its socioeconomic and racial/ethnic diversity, which is a byproduct of recruitment at a diverse array of clinical venues. Our cohort also includes a wide range of data from various sources—biological samples, questionnaires, and physical measurements from in-person visits, as well as electronic health records. We also have complete address histories for all NYU Langone participants that will allow us to geocode and use spatial mapping to assess spatially defined factors. Extant geospatial linkages include databases containing information on air pollution, noise, and neighborhood characteristics, including access to parks and healthy foods. The collection of specimens in three phases of pregnancy is another strength in that it allows us to examine trajectories of exposures over time and average them to account for variability due to non-persistence of certain chemical exposures. Our cohort is also well positioned to examine mixtures of non-persistent environmental chemicals at each time point in pregnancy and infancy. In addition, collection of infant samples is rare and permits examination of that window of vulnerability in relation to trajectories of *ex utero* growth.

Although we conceived of NYU CHES in the context of examining environmental exposures and child obesity, as part of the EWCP that is actively being implemented, our cohort is extremely well poised to examine neurodevelopmental and respiratory effects of prenatal and infant exposures to phthalates, bisphenols, OP pesticides, and PAHs, as well.

Selective non-response during the follow-up and retention are always challenges with an urban cohort, and the population of New York City is particularly fluid. Nonetheless, the comparison of mothers who provided consent to participate in the postnatal follow-up with those who decided not to (for reasons such as outmigration from New York City, lack of interest, or loss of contact with the study) confirms that participation in the postnatal phase of NYU CHES has not been selective. As shown in Table 8, participating mothers had similar age at enrollment, race/ethnic background, marital status, income, and employment status. Minor differences in characteristics such as education (higher educated mother participated in the follow-up), alcohol use (mothers who drank during pregnancy participated in the follow-up), and parity (participation rate of nulliparous mother was lower than parous mothers) will be considered in future analysis using appropriate epidemiological methods (e.g., inverse probability weighting).

**Table 8:** Descriptive characteristics of NYU CHES study participants by postnatal enrollment status

Characteristic^a^	Participants consented to postnatal phase	Participants not consented to postnatal phase
	n = 1597^a^	n = 403
Age at enrollment, mean (SD), years	32.0 (5.5)	31.2 (5.7)
Race/ethnicity, n (%)		
Hispanic	769 (49%)	196 (49%)
White, not of Hispanic origin	546 (34%)	131 (32%)
Black, not of Hispanic origin	84 (5%)	30 (7%)
Asian	145 (9%)	35 (9%)
Other	14 (1%)	3 (1%)
Multiple	35 (2%)	7 (2%)
Marital status, n (%)		
Married/living with a partner	1410 (88%)	347 (86%)
Divorced/separated	38 (3%)	6 (2%)
Single/widowed	149 (9%)	49 (12%)
Education, n (%)		
High school or less	491 (31%)	144 (36%)
Some college but no degree	187 (12%)	42 (11%)
Associate degree	76 (5%)	22 (6%)
Bachelor’s degree	360 (22%)	97 (24%)
Post graduate degree	472 (30%)	92 (23%)
Household income, n (%)		
Less than $30,000	258 (17%)	62 (16%)
$30,000–$49,999	128 (8%)	40 (11%)
$50,000–$74,999	104 (7%)	28 (7%)
$75,000–$99,999	70 (4%)	23 (6%)
$100,000 or more	630 (40%)	129 (34%)
Don’t know	368 (24%)	101 (26%)
Employed, n (%)	1046 (66%)	260 (65%)
Insurance, n (%)		
Public	798 (50%)	221 (55%)
Private	786 (50%)	175 (45%)
Use of cigarettes and other related products during pregnancy, n (%)		
Never	1450 (91%)	362 (90%)
Used, stopped in pregnancy	127 (8%)	35 (9%)
Used, continued in pregnancy	18 (1%)	5 (1%)
Alcohol use during pregnancy, n (%)		
Never	518 (32%)	144 (36%)
Used, stopped in pregnancy	809 (51%)	215 (54%)
Used, continued in pregnancy	268 (17%)	40 (10%)
Pre-pregnancy body mass index, mean (SD), kg/m^2^	26.4 (5.9)	25.8 (5.1)
Nulliparous, n (%)	779 (49%)	223 (56%)

^a^1597 mothers of 1624 children

Currently, the study collects information on fathers/partners through maternal reporting. These data provide valuable information including sociodemographic characteristics and weight and height, but the study remains limited in direct assessments of the fathers/partners as well as their evaluation of children’s growth and development. We also have not collected health histories or specimen from any parents biologically related to the child other than the birth mother.

## Collaboration

Regular data transmission to the NIH ECHO Data Analysis Center will permit centralized data analyses around common hypotheses, as well as preparation of de-identified and restricted datasets. For more information, see www.nih.gov/echo. For investigators interested in our primary data or in ancillary studies, NYU CHES has developed publications, specimen, and data sharing policies that are meant to encourage maximal use of this rich resource. More information can be obtained by contacting NYULHEnvPeds@nyulangone.org.
